# Exoscope and operative microscope for training in microneurosurgery: A laboratory investigation on a model of cranial approach

**DOI:** 10.3389/fsurg.2023.1150981

**Published:** 2023-03-24

**Authors:** Tommaso Calloni, Laura Antolini, Louis-Georges Roumy, Federico Nicolosi, Giorgio G. Carrabba, Andrea Di Cristofori, Marco M. Fontanella, Carlo G. Giussani

**Affiliations:** ^1^ School of Medicine and Surgery, University of Milano-Bicocca, Milan, Italy; ^2^ Neurosurgery, Fondazione IRCCS San Gerardo dei Tintori, Monza, Italy; ^3^ Neurosurgery, Department of Medical and Surgical Specialties, Radiological Sciences and Public Health, University of Brescia and Spedali Civili Hospital, Brescia, Italy

**Keywords:** exoscope 3D, microscope, training, education, residents, microsurgery, neurosurgeon surgical simulator, anatomical model

## Abstract

**Objective:**

To evaluate the viability of exoscopes in the context of neurosurgical education and compare the use of a 4k3D exoscope to a traditional operative microscope in the execution of a task of anatomic structure identification on a model of cranial approach.

**Material and methods:**

A cohort of volunteer residents performed a task of anatomical structure identification with both devices three times across an experimental period of 2 months. We timed the residents’ performances, and the times achieved were analyzed. The volunteers answered two questionnaires concerning their opinions of the two devices.

**Results:**

Across tries, execution speed improved for the whole cohort. When using the exoscopes, residents were quicker to identify a single anatomical structure starting from outside the surgical field when deep structures were included in the pool. In all other settings, the two devices did not differ in a statistically significant manner. The volunteers described the exoscope as superior to the microscope in all the aspects the questionnaires inquired about, besides the depth of field perception, which was felt to be better with the microscope. Volunteers furthermore showed overwhelming support for training on different devices and with models of surgical approaches.

**Conclusion:**

The exoscope appeared to be non-inferior to the microscope in the execution of a task of timed identification of anatomical structures on a model of cranial approach carried out by our cohort of residents. In the questionnaires, the residents reported the exoscope to be superior to the microscope in eight of nine investigated domains. Further studies are needed to investigate the use of the exoscope in learning of microsurgical skills.

## Introduction

Operative exoscopes (Exo) are a relatively novel class of devices for intraoperative visualization and magnification in Neurosurgery, consisting of an HD camera mounted to a support arm streaming images to a large screen. Multiple exoscope models are commercialized; the more advanced units are capable of producing 3D 4K images on large screens.

Superior ergonomics to traditional operative microscopes (OM) is often cited as one of the major, if not the biggest, advantages of the exoscope (1).

Abundant reports, albeit mostly based on surveys and opinions, can be found in the literature concerning the perceived educational value the use of the exoscope affords to trainees and expert surgeons alike (2, 3). With few exceptions (4), most of the educational value seems to be produced by equal view of the operative field by the surgeons and all the professionals in the operative room (5), generally superior to that of screens of traditional OMs, and the unhindered view of the lead surgeon’s hands (6), making it possible to teach to multiple residents at the same time.

Furthermore, conflicting data are reported concerning the learning curve of the exoscope across different settings, even in similar tasks (7, 8).

Few studies have comparatively investigated the use of the exoscope by residents for training purposes. During the execution of 2D and 3D tasks, a study found the traditional operative microscope to be superior to both 2D and 3D exoscopes and 3D exoscopes to be marginally better than 2D versions (9).

The aim of this work was to compare the learning curves of residents using an exoscope and an operative microscope to perform the same task, to investigate whether the new paradigm of exoscopic surgery could be suitable for a beginner, and to investigate how learning on the exoscope can be compared to that on the microscope.

## Methods

### Study objective

The primary objective of the study was to evaluate the use of an operative microscope (Leica OHX, Leica Biosystems) and a 3D exoscope (Orbeye, Olympus) by a cohort of residents during identification of anatomical structures.

In order to guarantee patient safety and repeatability, an anatomical model (UpSurgeOn, Milano) was used. The model reproduced a pterional approach, chosen because of its ubiquity and reproducibility in neurosurgical practice.

The primary objective of the study was to investigate if the improvement achieved by residents comfort.

Secondary objective of the study was the investigation of the residents’ progress over time with the two devices, whether and how seniority and previous experience influenced the performance of the single residents.

Finally, we sought to investigate how the study participants felt about the visualization devices and the use of anatomical models.

### Study design

The study was designed as a preclinical laboratory investigation with a group of volunteers using both instruments in an alternate fashion. All residents from University of Milano-Bicocca and University of Brescia Neurosurgery Residency programs who volunteered for the study were enrolled.

Prior to the first experimental session, investigators randomly generated 51 lists of 6 anatomical structures represented in the model of pterional approach to be used for the study. A so-called “Standard series” was generated from a subset of structures in the anterior cranial fossa and perisellar region. The small number and accessibility of the structures included in this series made it comparatively easier. The Standard Series is reported in Table 1.

**Table 1:** Structures in the randomization pool (A) and series resulting from the randomization (B).

A	B
L CN II	R M1
R CN II	R CN II
R ICA	R M1 bifurcation
R M1	R ICA
R M1 bifurcation	R A2
R A1	L A1
L A1	
ACoA	
L A2	
R A2	
Optic chiasm	

Fifty more series, numbered sequentially, were generated from a list including all the structures featured in the model. At the beginning of their first experimental session, each participant was randomly assigned of these series (henceforth referred to as “Personal Series”).

Two questionnaires were furthermore designed, one to be completed after each experimental session and one after the last session (Tables 2, 3).

**Table 2:** Investigated domains, answer types, and answers to questionnaire 1.

Item	Question	Answer type	Results
1	Compare: overall image quality	Likert-type scale 0–10 0 = OM markedly superior 5 = comparable 10 = Exo markedly superior	Average score: 6.47
2	Compare: brightness		Average score: 6.60
3	Compare: ergonomics		Average score: 7.97
4	Compare: mobility		Average score: 8.14
5	Compare: depth of field perception		Average score: 4.6
6	Compare: ease of focus		Average score: 7.68
7	Compare: control of movement		Average score: 7.25
8	Compare: overall ease of use		Average score: 7.12
9	Rate: perceived discomfort during Exo use	Likert-type scale 0–10	Average score: 3.10
10	How many total procedures did you take part in?	a) <10 b) ≥10, <50 c) ≥50, <200c	See Figure 3
11	How many exoscopic procedures did you take part in?		
12	Have you more than 100 h of lifetime videogaming?	Y/N	Y = 12 (75%) N = 4 (25%)

Exo, exoscope; OM, operative microscope.

**Table 3:** Investigated domains, answer types and answers to questionnaire 2.

Item	Question	Answer type	Results
1	Which device was globally easier to use?	Exo/OM	Exo: 15 (93.7%) OM: 1 (6.7%)
2	Which device had you more experience using prior to this training?	Exo/OM	OM: 16 (100%)
3	Do you believe skills acquired with one device translate well to the other?	Y/N	Y: 5 (31%) N: 11 (69%)
4	Do you believe the Exoscope to require specific training?	Y/N	Y: 6 (37.5%) N: 10 (62.5%)
5	If you were asked to choose a single system to use (after specific training), which one would you pick?	Exo/OM	Exo: 12 (75%) OM: 4 (25%)
6	Which device feels more intuitive?	Exo/OM	Exo: 12 (75%) OM: 4 (25%)
7	Which device do you believe will be the gold standard in the future?	Exo/OM	Exo: 14 (87.5%) OM: 2 (12.5%)
8	How useful did you find taking part in this study to be for your education?	Likert-type scale 0–10	Average score: 7.875
9	How useful was taking part in this study for your ability to orient yourself in pterional anatomy?	Likert-type scale 0–10	Average score: 7.88
10	How useful do you believe training on models to be during neurosurgical education?	Likert-type scale 0–10	Average score: 9.5

Exo, exoscope; OM, operative microscope.

**Figure 1:**
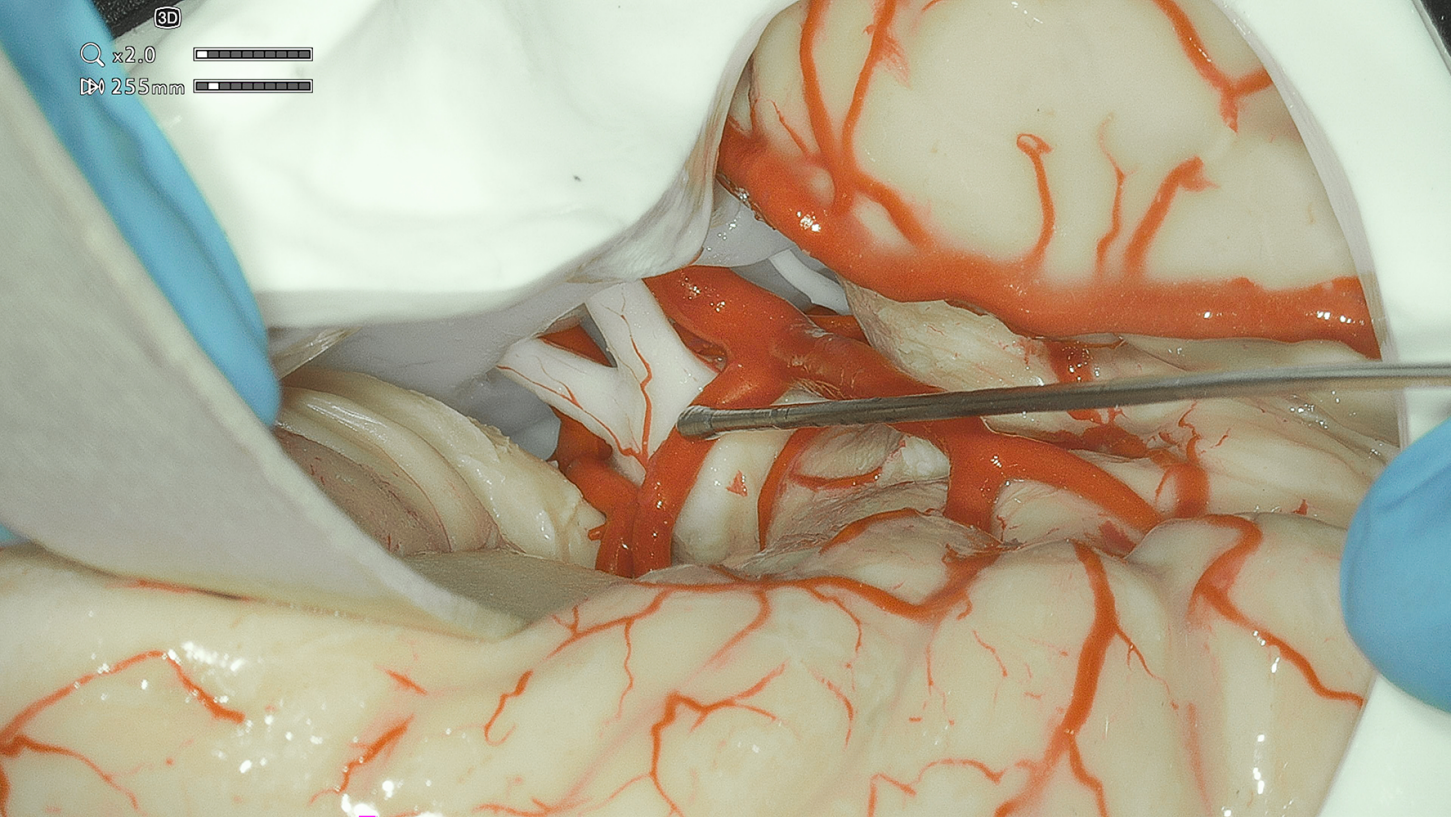
Exoscope-acquired picture of the anatomical model used in the study, depicting some of the structures included in the standard series.

Prior to the study, all participants received specific instructions concerning the aims and modality of the study and a brief lecture concerning the pterional approach and relevant topographic neuroanatomy. Furthermore, the participants were given pictures of the anatomical model (Figure 1). Before each session, participants were allowed some time to refamiliarize themselves with both the model and the devices.

**Figure 2:**
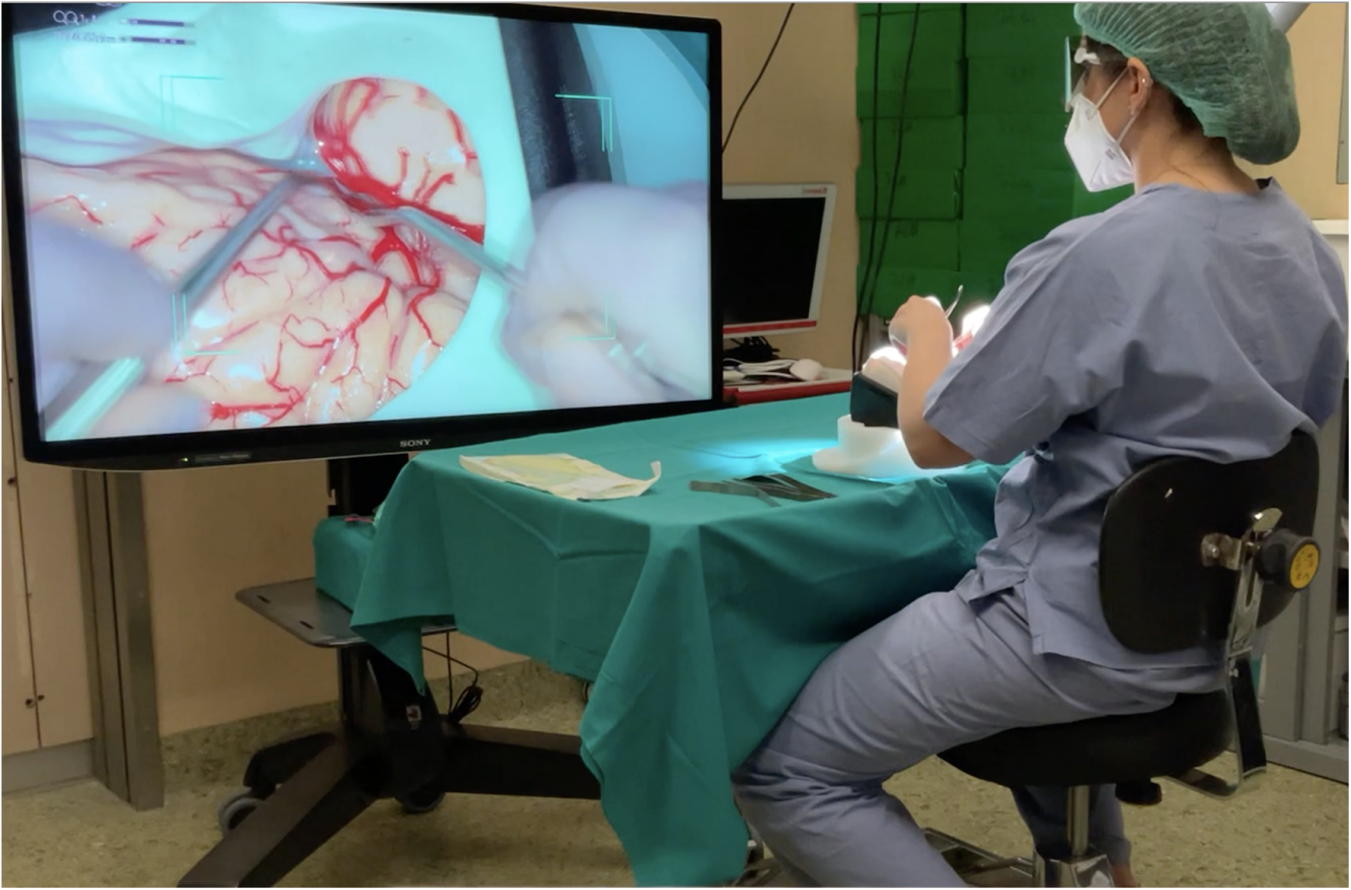
Image from Supplementary Video 1 depicting the experimental setup during task execution with the exoscope.

Multiple surgical instruments, including metal suction tubes, spatula, and tweezers, were provided, and the participants were free to choose which instruments to use. The surgical model was placed on a table, and the participants were sitting on a chair, set to a comfortable height by each participant prior to starting the task (Figure 2).

At the beginning of the task, the visualization device was in the resting position pointing next to the operative field. The participants were required to use the visualization device to identify each structure sequentially, and identification was performed by touching each structure and giving voice confirmation.

After each structure was identified correctly, the investigator named the subsequent structure in the series.

On each experimental session, participants went through the Standard series and the Personal series they were assigned with each visualization device.

The participants were not informed that the same two sets of structures (Standard and Personal Series) would be repeated each time. Across sessions, the order in which each participant used the devices were used was alternated to compensate for a possible warm-up effect.

The task was timed, and the timer was started when the investigator named the first structure to be identified and stopped when the sixth and last structure in the series was identified. The interval to each structure was also measured. Times were recorded in seconds and rounded to the nearest whole (for a video of task execution, see Supplementary Material 1).

For analysis purposes, times to the first structure and times from the second to the sixth structure were considered as separate groups to account for the need to position the visualization device over the operative field.

### Questionnaires

After each session, the participants completed a questionnaire (Questionnaire 1). They were asked to complete a second questionnaire (Questionnaire 2) at the end of the study. The questionnaires were designed to gather information concerning the previous experience of the participants with the visualization device, their assessment concerning multiple domains of the visualization device, and their preferences and opinions.

Questionnaire 1: Items 1 through 8 required the residents to score different aspects of the exoscope as compared to the microscope on a Likert-type scale of 1 (very inferior) to 10 (very superior). Higher scores underlie and perceived superiority of the exoscope. Item 9 investigated discomfort and optic strain when using the exoscope, a score between 1 (absent) and 10 (unbearable) was given by each participant.

Items 10 and 11 inquired about the amount of the participant’s previous experience with both devices. Item 12 inquired about whether the resident had significant amounts (arbitrarily chose as 100 h) of videogaming experience (see Table 2).

Questionnaire 2: This questionnaire investigated multiple domains of participants’ opinion of the two visualization devices, their personal preference, and whether they found training similar to the experimental design useful (questions are reported in Table 3).

### Statistical analysis

The distribution of the time to identify the first structure was described by mean and compared by a standard *T*-test. The total time was calculated by summing up the times from structures 2 to 6. The distribution of the total time was described in each session and level of experience by mean, SD, and quartiles, and represented in boxplots. The impact on the total time of the type of device, time (through sessions), and level of self-reported experience was assessed by a general linear mixed model with a random intercept. Statistical analysis and graphics were carried out by Stata Software version 16.

## Results

Between March and May 2022, 16 residents (see Table 4) from the University of Milano-Bicocca and the University of Brescia performed the whole circuit three times. Thirteen total experimental sessions were necessary based on volunteers’ and examiners’ availability, each session lasting between 1.5 and 3.5 h. The average interval between each session for all participants was 22.03 days (median: 21 days).

**Table 4:** Demographic data of the volunteers.

Gender	Males	7 (43.75%)
	Females	9 (56.25%)
Seniority	PGY1	8 (50%)
	PGY2	3 (18.75%)
	PGY3	2 (12.5%)
	PGY4	2 (12.5%)
	PGY5	1 (6.25%)
Self-reported experience (no. of surgeries)	<10	4 (25%)
	≥10 and <50	7 (43.75%)
	≥50	5 (31.25%)
University	Milano-Bicocca	12 (75%)
	Brescia	4 (25%)

### Standard series

#### First structure

No statistical difference was thus observed in the average time necessary for identification of the first structure between the microscope (15.4 s) and the exoscope (14.3 s) (*p*: 0.552) on the first try. A statistically significant improvement in the speed with each device was observed on the second and third tries, on average at 12.4 s (*p*: 0.008) and 10.3 s (*p*: 0.0001), respectively, on the microscope, and 9.5 s (*p*: 0.0001) and 10.5 s (*p*: 0.004), respectively, with the exoscope. Improvement between the second and third tries approached statistical significance on the microscope (*p*: 0.056), while it was not on the exoscope (*p*: 0.455). The average time on the final try did not differ between the exoscope and the microscope (10.5 and 10.3 s, *p*: 0.78).

#### Second to sixth structures

The average time required for task performance diminished significantly across the experiment. With the microscope, it went from 27 to 20.563 s (*p*: 0.014) on the second try to 18.875 s (*p*: 0.002) on the third try. Improvement between the second to the third try was not statistically significant (*p*: 0.52).

On the first try with the exoscope, the average time required was 26.06 s. This later improved (i.e., shortened) to 19.81 s (*p*: 0.003) and 16.12 s, respectively. Again, improvement from the second to the third try failed to prove significant (*p*: 0.08).

The average time with the two devices did not differ significantly at any point during the experiment.

### Personal series

While the series varied across participants, each participant was tested on the same each time, making it possible to compare aggregate performances.

#### First structure

Participants averaged 20.983 s using the microscope and 14.375 s using the exoscope. While using the microscope, the residents improved their performance significantly on both the second (15.25 s, *p*: 0.009) and the third tries (14.875 s, not statistically significant compared to the second try, *p*: 0.86).

Using the exoscope, the performance improvement in average time was not statistically significant on the second try at 12.75 s (*p*: 0.20); on the third (time: 10.56 s), the change was not significant compared to the second (*p*: 0.087), but it was compared to the first (*p*: 0.003).

This was the only instance that demonstrated a significant difference between instruments: the exoscope fared significantly better on the first (*p*: 0.01) and third tries (*p*: 0.001) compared to the microscope.

#### Second to sixth structures

The average time needed for the completion of the task with the microscope was 36.03, 26.5, and 24.87 s on the first, second, and third executions, respectively, with a statistically significant improvement between the first and second execution (*p*: 0.01) and not between the second and the third (*p*: 0.66).

The improvement was observed also with the exoscope, with an average time on the first execution of 40.9 s, which later improved to 27.62 and then 21.87 s. The improvement proved significant between the first and second attempt (*p*: 0.006) but not between the second and third (*p*: 0.235).

The final times achieved with the exoscope and the microscope did not display statistically significant differences (*p*: 0.27).

### Standard series results and self-reported surgical experience

We furthermore looked at the average times achieved by residents to identify structures 2 through 6 of each series and evaluated whether it was correlated with the self-reported previous surgical experience.

**Figure 3:**
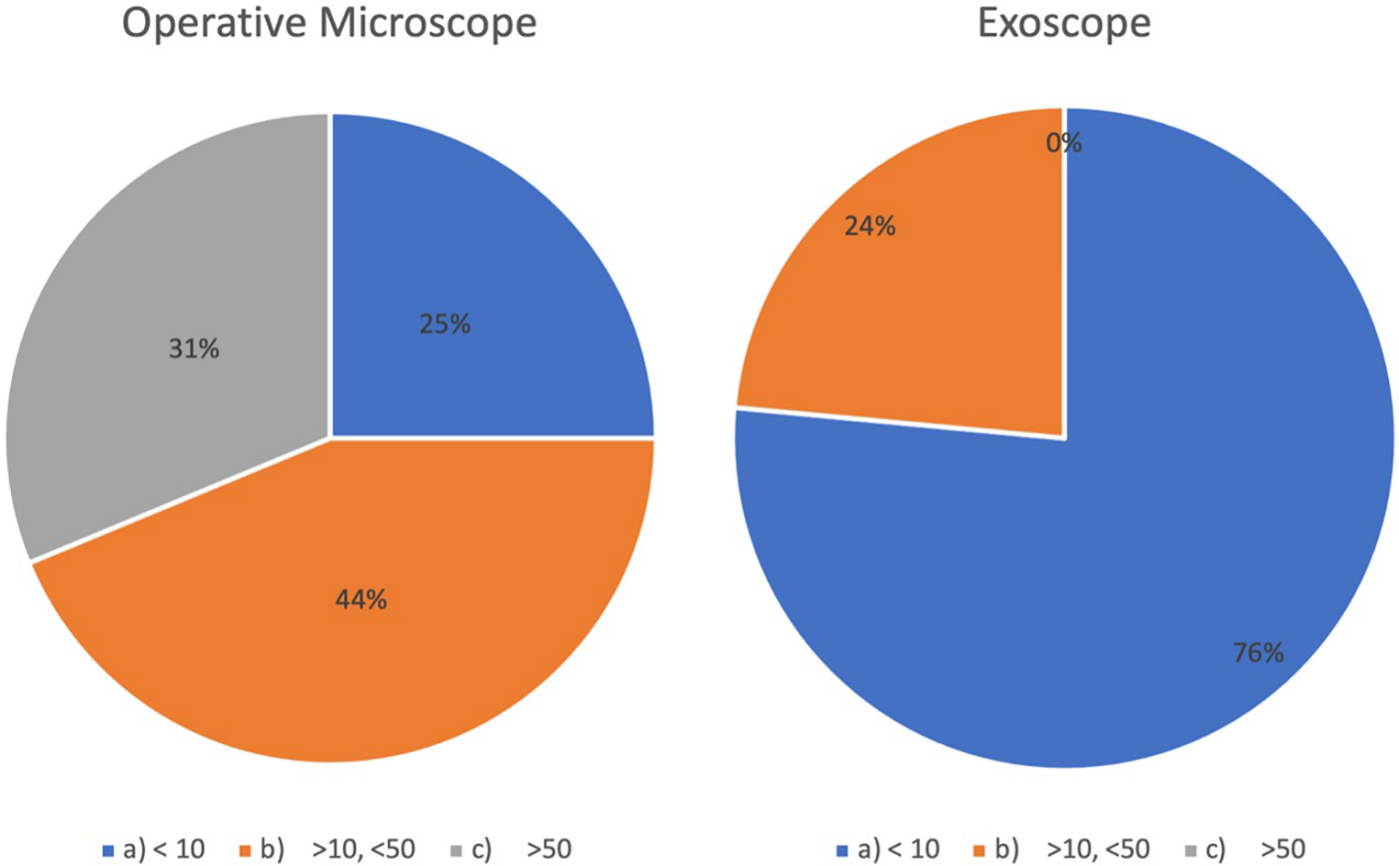
Microsurgical experience (number of surgeries) prior to the start of the study reported by volunteers based (answers to questions 10 and 11 of questionnaire 1) with the OM and the exoscope. OM, operative microscope.

Residents were classified in group A (less than 10 surgeries), B (10–49 surgeries), and C (more than 50 surgeries) based on their answer to Item 10 of Questionnaire 1 (see Figure 3). As residents from different university networks and hospitals were included in the study, we believed the amount of surgeries performed at the beginning of the study to be a more comparable parameter than Post-Graduate Year (PGY).

**Figure 4:**
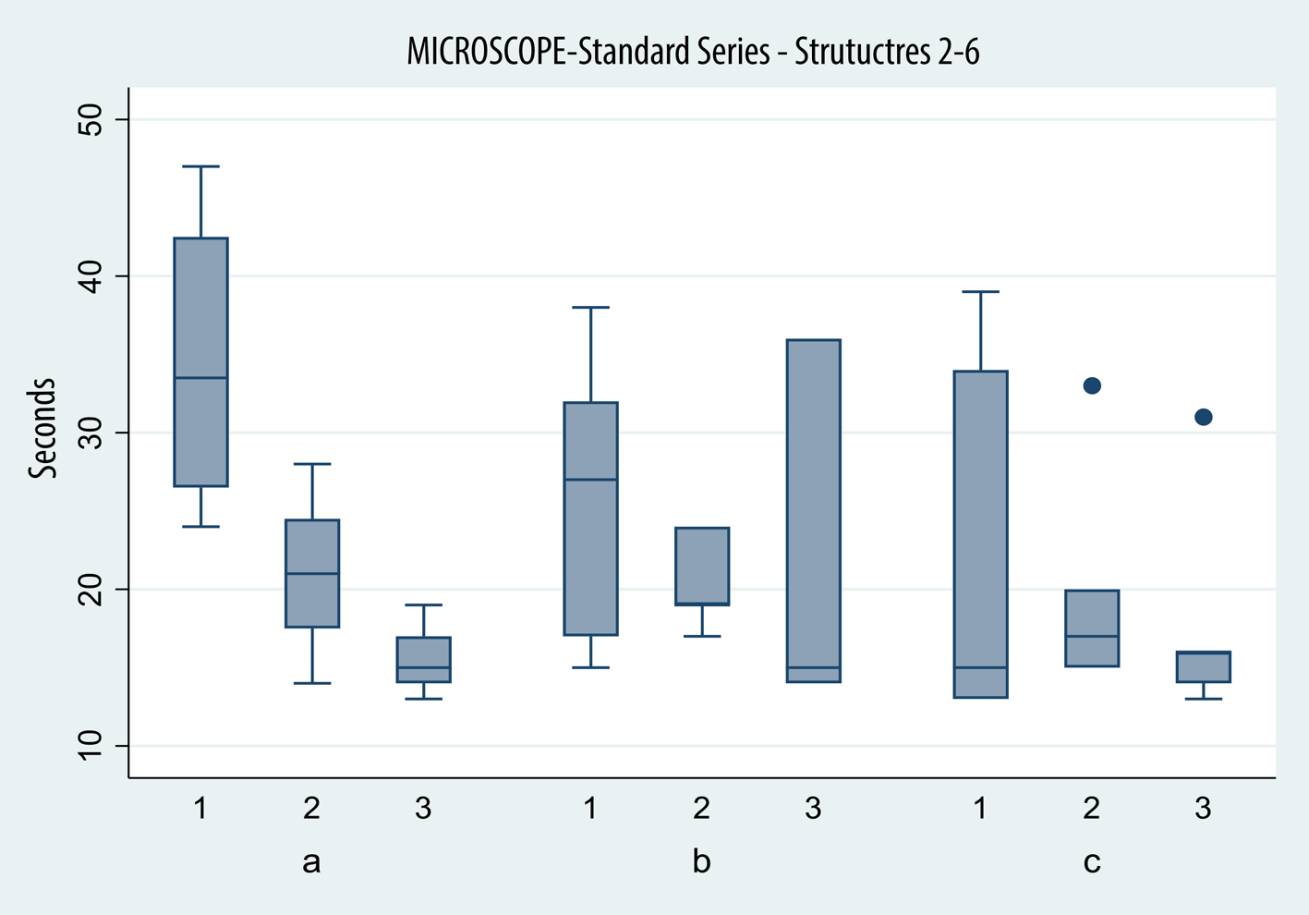
Box plot depicting times in seconds (*y* axis) achieved with the microscope in identifying structures 2–6 of the standard series on successive tries (*x* axis) by the three groups (a, beginners; b, intermediate; and c, advanced).

**Figure 5:**
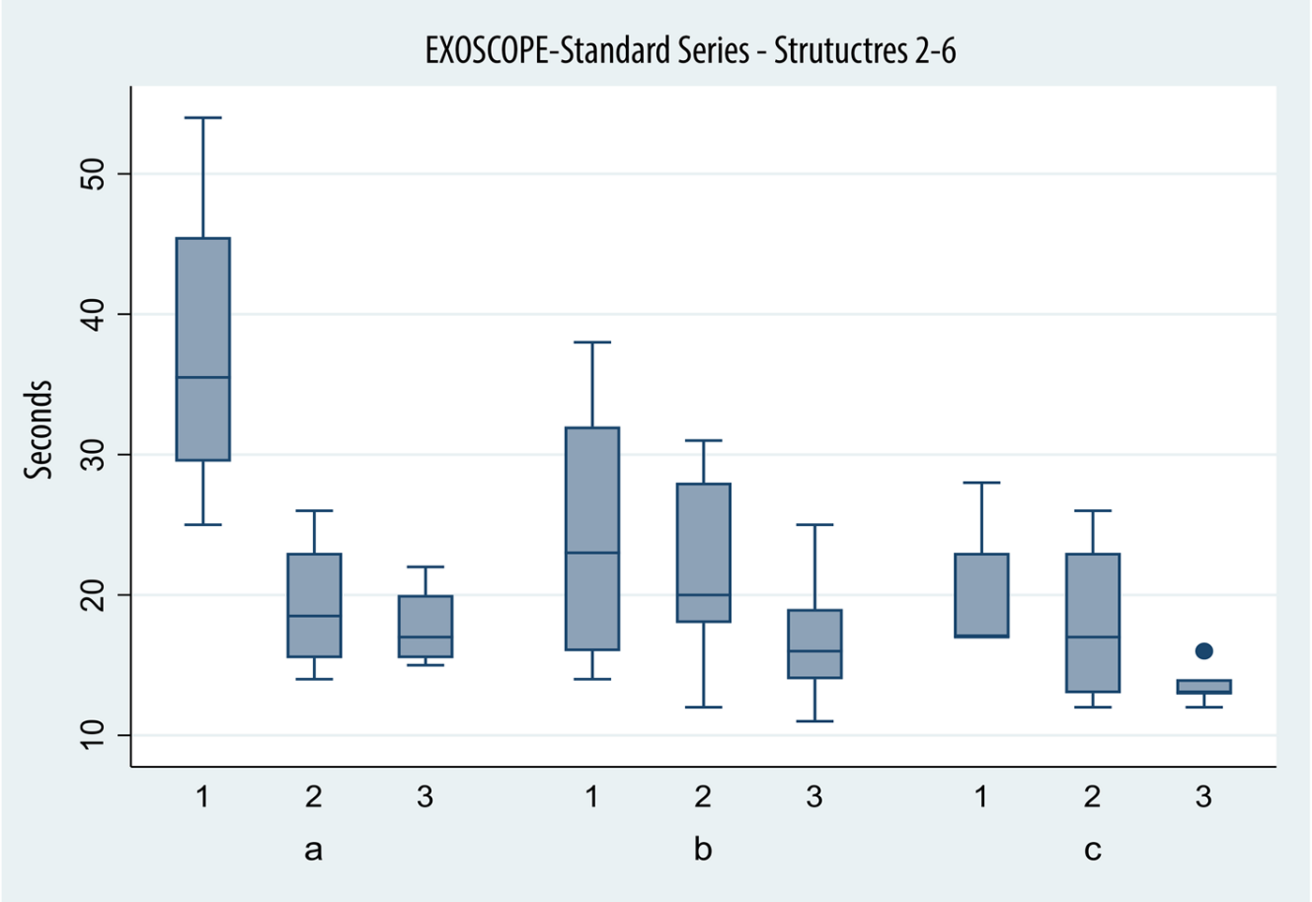
Box plot depicting times in seconds (*y* axis) achieved with the exoscope in identifying structures 2–6 of the standard series on successive tries (*x* axis) by the three groups (a, beginners; b, intermediate; and c, advanced).

The recorded times across attempts and devices of the three groups are represented in Figures 4, 5.

#### Group A (beginners: <10 surgeries) (*N*: 4)

Group A achieved average times of 34.5, 21.0, and 15.50 s on the microscope, displaying a significant improvement between the first and second (*p*: 0.01) sessions and not between the second and third (*p*: 0.192).

On the exoscope, the results were similar: 37.5 s on the first try, 19.50 s on the second, and 17.75 s on the third try. Again, the improvement proved to be significant between the first and second attempt (*p*: 0.00) and not between the second and third (*p*: 0.75).

#### Group B (intermediate: ≥10 and <50 surgeries) (*N*: 7)

Average times for task completion with the microscope were 25.71, 20.71, and 21.43 s across the first, second, and third attempts by group B. The improvement failed to prove statistical significance across sessions (*p*: 0.2 and *p*: 0.855).

Average time was 23.57 s on the first session, 21.29 on the second, and 17 s on the third, when using the exoscope. The improvement proved to be significant only between the first and third sessions (*p* > 0.0001).

#### Group C (advanced ≥50 surgeries) (*N*: 5)

Microscope times were 22.80, 20, and 18 s, respectively, across three attempts. Again, the improvement, while found, failed to cross the threshold to statistical significance (*p*: 0.453, *p*: 0.59).

The times recorded when the exoscope was used were 20 and 40 s, then 18.20 and 13.60 s respectively. The difference was not significant between the first and second attempt (*p*: 0.27) and the second and last (*p*: 0.02), but it was between the first and third (*p* < 0.0001).

The average time on the last session was 18 s with the microscope and 13.6 s on the last session; the difference in results approached statistical significance (*p*: 0.07).

### Experience group comparison

On the first try with the microscope (groups A, B and C), the difference in times, while present, was not statistically significant, even when comparing group A and group C (*p*: 0.116).

A different effect was observed only during exoscope use, when comparing group A and B (*p*: 0.025), with more experienced residents faring significantly better, not between intermediate and experienced residents (*p*: 0.54).

By the third try, no statistically significant difference was observed in the speed of the three groups with either device.

### Videogame experience

We compared two subgroups based on Item 12 of questionnaire 2, which inquired whether the participant had played video games for more than 100 h, and no statistically significant differences emerged.

Furthermore, we evaluated whether gamers fared better with the exoscope than the microscope; again, no statistically significant differences were found.

### Questionnaire results

#### Questionnaire 1

Investigated domains and scores are reported in Table 2.

#### Questionnaire 2

The exoscope was considered to be easier to use by 93.7% of the residents (Item 1).

All of the residents reported having used the microscope more than the exoscope before this study (Item 2). Among the participants, 31.2% believed the experience acquired with one device would translate to the other (Item 3).

Specific training prior to exoscope use was believed to be necessary by 37.5% of the participants (Item 4).

If forced to adopt only one device, 75% would pick the exoscope (Item 5).

The exoscope was considered to be more intuitive to start using by 75% of the respondents (Item 6).

87% for the volunteers believed the exoscope will be adopted as the standard visualization device in the Operatory Room (OR) in the ftuture (Item 7).

Residents were asked to report how useful they considered the activities carried out in this study to be for their own education concerning the use of different visualization devices (Item 8), the average score was 7.875 (median: 7.8), and concerning how useful they considered the activities carried out in this study to be for their own education fort their spatial orientation and anatomic understanding, the average score was 7.88 (median: 8) (Item 9).

Training on anatomic models was strongly praised by residents, with an average score of 9.5 (median: 10) on a scale of 1–10 (Item 10).

## Discussion

While extensive speculation exists concerning the educational value of the exoscope as compared to the operative microscope (3), evidence of this advantage is mostly based on surveys and expert opinions (6, 10).

In this study, as we expected, the participants’ performance, assessed as time required to carry out the task, improved over time, i.e., they became progressively more efficient in structure identification. This effect was observed with both the exoscope and the OM and both on the so-called *Standard* and *Personal series*.

When the participants were considered together, the times achieved across three sessions in both the identification of the first structure and structures 2–6 do not differ significantly based on the device in use: the improvement occurs thus in parallel between instruments according to the pooled performance of all residents in this study. The only exception is the identification of the first structure in the personal series: the time to identify the first structure starting from a resting position outside the field was significantly longer with the microscope than with the exoscope, likely an effect of the superior maneuverability of the latter. This effect was maintained over time.

The improvement in the second to sixth structures’ identification was apparent with both instruments, generally more marked between the first and second attempt than between the second and the third. In the Special series, the more challenging of the two, the improvement was more marked with the exoscope.

While this could be due to lower starting ability with the exoscope and, thus, bigger margins for improvement with the exoscope, the average times at the beginning of the experiment fail to demonstrate a significant difference, which contradicts this theory. Instead, we propose the effect is due to the exoscope being better suited for tasks involving rapid readjustment among different, somewhat distant, structures.

Concerning the effects of previous experience, all the residents reported greater experience in using the microscope compared to the exoscope prior to this study (Questionnaire 2, Item 2), but this did not seem to contribute to any significant advantage in using the microscope compared to the exoscope when the whole group was considered.

### Experience subgroups analysis

#### Microscope

In the experience subgroup analysis, an effect of the previous amount of experience was observed but failed to prove statistically significant.

Only the beginner group was able to achieve significant improvement over each try; both the intermediate and advanced group failed to do so. We believe this is a consequence of the more experienced groups having already achieved a good level of proficiency in the use of the microscope prior to enrollment in this study. Indeed, the performance of the latter groups is better than those of beginners when starting out.

Unsurprisingly, greater microscopic surgery experience seems to correlate with better performances in this task, but less experienced residents were able to rapidly catch up to their colleagues, while margins for further optimization by experienced subjects seem slim.

#### Exoscope

The beginner group fared significantly worse on the first try than both the intermediate and advanced groups.

The performance of the beginner group markedly improved on the second try, achieving a time comparable to that of more experienced colleagues’ first try. The intermediate group, while improving across tries, failed to do so in a statistically significant manner from each to the next. The advanced group did not display significant improvement from the first to the second try and last try either.

The data suggest that previous experience, even with the microscope, proves to be useful when starting to use the exoscope, but this effect again disappears rapidly (i.e., the learning curve is not very steep).

Results on the first try for the advanced group did not differ significantly between the exoscope and the microscope, but there was a trend toward a significant difference on the last try (*p*: 0.07). A possible explanation in the exoscope is better suited for such a task than the microscope, possibly because identifying out of field structures requires only readjustment of the camera and not the user’s upper body, as does the microscope. Thus, the performance ceiling is higher with the exoscope than the microscope, but it is only accessible to relatively more experienced users.

This is in contrast with the evidence in the literature of a superiority in 2D and 3D tasks by the OM compared to the 3D exoscope (9).

Concerning the use of videogames, this requires and somewhat trains visual attention, hand–eye coordination, and depth perception, which are relevant skills for surgeons (11, 12). Some authors have speculated that the lack of videogame experience could be a factor in preferring the microscope to the exoscope (13). In our group, the ones who denied videogaming experience did not show meaningful differences in performances compared to their peers.

In Questionnaire 1, residents scored the exoscope to be superior to the microscope in all the aspects inquired about except the perception of field depth. Indeed, depth perception has been reported as a significant drawback in multiple (14, 15) reports, although on the other hand, large depth of field has been praised as an advantage by other authors (16) and so do our senior authors.

Another pitfall of the exoscope is the reported eyestrain (13). In our group, the average reported eyestrain during exoscope use was 3.10 on a scale of 1–10. While the task execution only took a short time, the task consisted in rapid movement of the exoscope, which might have exacerbated this feeling for some.

Concerning Questionnaire 2, the residents in this study demonstrated overwhelming appreciation for the exoscope and a large majority believe it will become the standard visualization device in Neurosurgery. Of note, only a minority believe the training received on one device translates well to the other, while in our study, cumulative prior experience correlated with performance across groups: residents with comparable cumulative experience performed similarly with both devices event though they reported to have more experience using the OM.

Participants in this study furthermore described activities such as those carried out for the study to be very useful for both anatomical and device-specific education and expressed overwhelming support for the use of anatomical models for teaching.

Limitations of this study include the small sample size, the large percentage of junior residents (PGY1) enrolled, the chosen task, and the crossover design, with the possibility of skill improvement being carried over among instruments.

Future perspectives in this topic concern investigation of the educational value for residents and junior surgeons in the learning of surgical skills.

## Conclusion

We evaluated the performance of a group of residents in various stages of their neurosurgical education in a task 3D HD exoscope. The exoscope appeared to perform better when used to identify deeper structures while starting from outside the surgical field; otherwise, neither device seemed to offer significant advantages compared to the other in the execution of the task when the whole group was considered.

Interestingly, our population seemed more inclined toward the exoscope than the microscope and scored the exoscope higher in all domains but depth perception. Further studies are needed to clarify the role and possible advantages of the exoscope in residents’ education.

## Data Availability

The raw data supporting the conclusions of this article will be made available by the authors, without undue reservation.
